# A Review on Preventing Tragedy: Strategies to Combat the Devastating Effects of Adolescent Drug Overdoses

**DOI:** 10.7759/cureus.39132

**Published:** 2023-05-17

**Authors:** Gaurav Sharma, Swarupa Chakole, Roshan Prasad, Mayur B Wanjari, Ranjana Sharma

**Affiliations:** 1 Medicine, Jawaharlal Nehru Medical College, Datta Meghe Institute of Higher Education and Research, Wardha, IND; 2 Community Medicine, Jawaharlal Nehru Medical College, Datta Meghe Institute of Higher Education and Research, Wardha, IND; 3 Medicine and Surgery, Jawaharlal Nehru Medical College, Datta Meghe Institute of Higher Education and Research, Wardha, IND; 4 Research and Development, Jawaharlal Nehru Medical College, Datta Meghe Institute of Higher Education and Research, Wardha, IND; 5 Medical Surgical Nursing, Smt. Radhikabai Meghe Memorial College of Nursing, Datta Meghe Institute of Higher Education and Research, Wardha, IND

**Keywords:** adolescents, drug overdose, opioid epidemic, policy changes, treatment and support services, risk factors, prevention strategies

## Abstract

Adolescent drug overdose deaths are a growing public health crisis, with significant consequences for individuals, families, and communities. This review article provides a comprehensive overview of prevention strategies to combat the devastating effects of adolescent drug overdose. Drawing on a comprehensive literature search of electronic databases, the article evaluates the effectiveness of prevention strategies and identifies risk factors associated with overdose deaths. The review outlines three key prevention strategies, including education and awareness programs, access to treatment and support services, and policy changes and regulations. The article also discusses the limitations and challenges of prevention efforts, including limited access to treatment and support services, the need for more research on effective prevention strategies, and the ongoing challenges posed by the opioid epidemic and the emergence of new synthetic drugs. Overall, this review highlights the urgent need for continued research, innovative prevention strategies, and effective policies to prevent adolescent drug use and overdose deaths and promote healthier communities for all.

## Introduction and background

Adolescent drug overdose deaths represent many countries' significant and growing public health crises. According to the National Institute on Drug Abuse (NIDA), there has been a significant increase in drug overdose deaths among adolescents and young adults over the past two decades. Additionally, NIDA reports that drug overdose deaths involving stimulants, cocaine, or psychostimulants with abuse potential (primarily methamphetamine) have significantly increased since 2015, rising from 12,122 deaths to 53,495 deaths in 2021 [1]. The most commonly implicated drugs in these tragic deaths include prescription opioids, synthetic opioids, heroin, and benzodiazepines. Factors contributing to this trend include the ease of access to prescription medications, the increased use of illicit drugs, and a lack of awareness and education on the dangers of drug use [2,3].

Preventing adolescent drug overdose deaths is essential for promoting the health and well-being of our youth. Adolescents who misuse drugs are at a higher risk of developing addiction, mental health issues, academic problems, and social and legal consequences. Effective prevention strategies are crucial in mitigating these risks and preventing further tragedies [3]. Furthermore, investing in prevention strategies can save lives, reduce healthcare costs, and improve overall community health. It is therefore imperative to identify and implement effective prevention strategies to address the growing problem of adolescent drug overdose deaths [1,3].

The review article aims to provide a comprehensive overview of the current state of adolescent drug overdoses and the impact of this public health crisis on individuals, families, and society. The article aims to examine various prevention and intervention strategies to combat the devastating effects of adolescent drug overdoses, including evidence-based prevention programs, access to treatment and recovery services, education and awareness campaigns, and policy initiatives. Ultimately, the article aims to provide valuable insights and recommendations for stakeholders, policymakers, and healthcare professionals to develop and implement effective strategies to prevent and address adolescent drug overdoses.

## Review

Methodology

**Figure 1:**
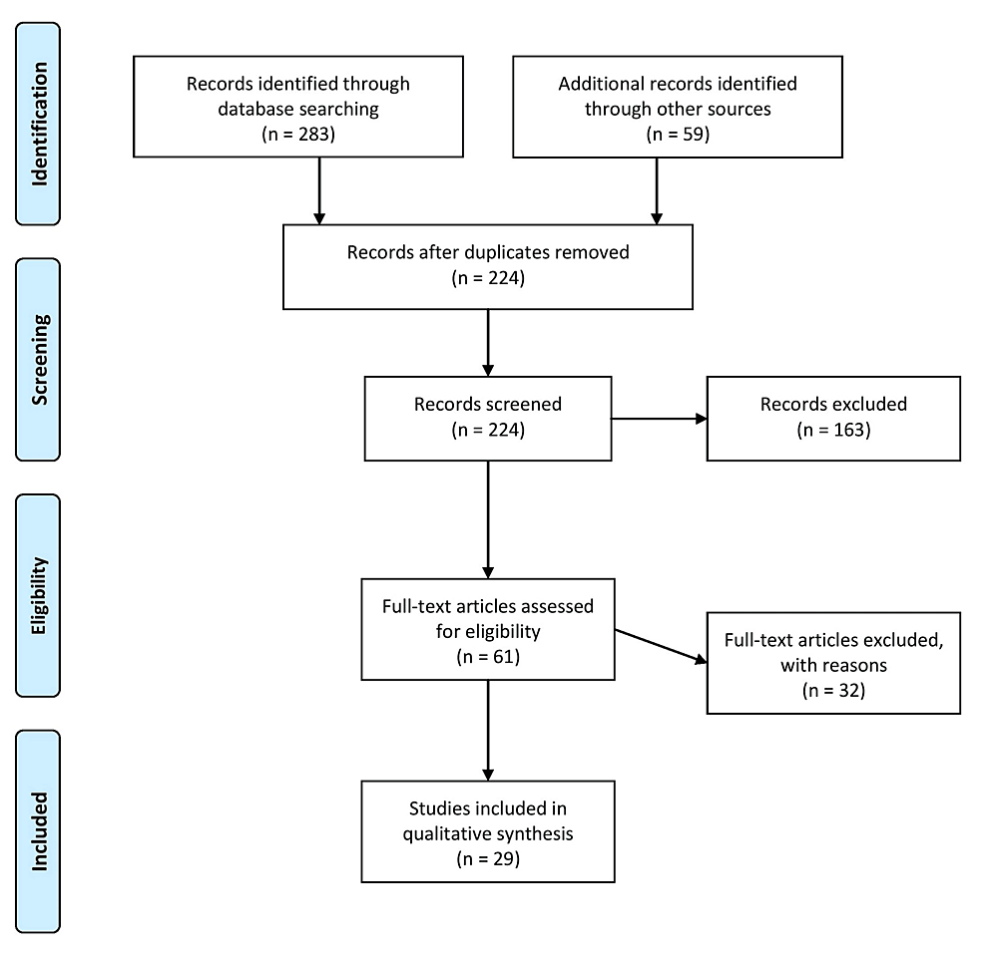
The selection process of articles used in this study Adopted from the Preferred Reporting Items for Systematic Reviews and Meta-Analyses (PRISMA)

A comprehensive literature search was conducted using electronic databases such as PubMed, Medical Literature Analysis and Retrieval System Online (MEDLINE), PsycINFO, and Cochrane Library. The search encompassed articles published from 2000 to the present. The search was conducted using Medical Subject Heading (MeSH) terms. It utilized specific keywords such as "adolescents," "drug overdose," "prevention strategies," "treatment programs," "policy changes," and "risk factors." Articles published between 2000 and 2023 were included in the review. After the initial search, articles were screened based on their relevance to the topic and inclusion criteria. The inclusion criteria included articles that evaluated prevention strategies for adolescent drug use and overdose, provided data on the effectiveness of prevention strategies, and identified risk factors for overdose deaths. The inclusion criteria needed them all to be present in a selected article. The exclusion criteria included articles that focused solely on adult populations, were not published in English, or were not peer-reviewed. Figure 1 describes the selection process of articles used in our study.

Risk factors for adolescent drug overdose deaths

Common Substances Involved in Overdose Deaths

Adolescents who misuse drugs are at a heightened risk of experiencing an overdose, which can ultimately result in severe health complications or death [4]. Among the substances commonly associated with drug overdose deaths among adolescents are prescription opioids, synthetic opioids, heroin, and benzodiazepines [5]. These drugs have been identified as major contributors to respiratory depression, which can ultimately lead to death. Given the potency of these drugs, it is crucial that adolescents are educated about their risks and that preventative measures are taken to minimize their misuse [2,4]. The common substance associated with overdose death in India is mentioned in Table 1.

**Table 1:** Common substances involved in overdose deaths in India The author has created a table from the source [4-9] MDMA: 3,4-methyl​enedioxy​methamphetamine

Substance	Common Forms	Overdose Symptoms
Opioids	Prescription painkillers, heroin, and synthetic opioids (fentanyl)	Extreme drowsiness, slowed breathing, loss of consciousness, and pinpoint pupils
Benzodiazepines	Prescription sedatives and antianxiety medications (Xanax and Valium)	Confusion, dizziness, slowed or difficulty breathing, and loss of coordination
Cocaine	Powdered form (cocaine hydrochloride) and crack cocaine	Increased heart rate, high blood pressure, chest pain, and seizures
Methamphetamine	Crystal meth and meth powder	Increased energy, rapid heartbeat, paranoia, and hallucinations
Alcohol	Beer, wine, and spirits	Slurred speech, impaired coordination, blackouts, and unconsciousness
Synthetic cannabinoids	Spice and K2	Severe agitation, anxiety, hallucinations, and rapid heart rate
Ecstasy (MDMA)	Pills and tablets	Increased energy, elevated body temperature, and rapid heartbeat
Synthetic opioids	Fentanyl and carfentanil	Respiratory depression, unconsciousness, and pinpoint pupils
Barbiturates	Prescription sedatives (phenobarbital and secobarbital)	Drowsiness, confusion, slowed breathing, and coma
Inhalants	Household chemicals (paint thinner, gasoline, and aerosol sprays)	Dizziness, euphoria, confusion, and cardiac arrest

Underlying Mental Health Conditions

Mental health conditions, including but not limited to depression, anxiety, and trauma, frequently underlie drug use and overdose among adolescents [6]. Adolescents who struggle with mental health issues may use drugs to self-medicate or cope with their symptoms. For example, some adolescents with depression may turn to drugs to alleviate feelings of hopelessness or despair, while others with anxiety may use drugs to ease tension or nervousness [7]. Adolescents who have experienced trauma may also use drugs to numb painful emotions or memories associated with their traumatic experiences. Therefore, addressing mental health issues is critical in preventing adolescent drug use and overdose [8].

Peer Pressure and Social Influences

Peer pressure and social influences can exert a significant influence on adolescent drug use and overdose [9]. Adolescents may feel compelled to conform to their peer group and gain acceptance by experimenting with drugs. The desire to impress peers or gain social status can also motivate adolescent drug use. Moreover, exposure to drug-using peers or a lack of positive role models can increase the likelihood of drug use and subsequent overdose. Such influences can result in adolescents adopting risky behavior patterns that can lead to serious health consequences, including addiction, overdose, and even death [10,11].

Lack of Parental Supervision and Support

Adolescents who do not receive adequate parental supervision and support may be at a greater risk of engaging in risky behaviors such as drug use. Parents play a crucial role in reducing the likelihood of drug use and overdose by providing guidance, establishing clear expectations and boundaries, and promoting positive decision-making skills and behaviors [12].

A lack of parental supervision and support can leave adolescents feeling disconnected and vulnerable, potentially leading them to seek validation and connection through peer relationships, increasing the risk of drug use. Furthermore, adolescents without parental guidance may not have access to important information about the dangers of drug use or the skills needed to resist peer pressure to use drugs [13].

Research has consistently shown that parental involvement and support are associated with lower drug use and adolescent overdose rates. This involvement can take many forms, such as establishing open lines of communication, monitoring adolescents' behavior, and setting clear expectations and boundaries around drug use. Additionally, parental support can include emotional support, promoting healthy behaviors, and providing access to mental health and substance abuse treatment services when needed [9,12,13].

Strategies for preventing adolescent drug overdose deaths

Education and Awareness Programs

School-based prevention programs: School-based prevention programs offer a valuable opportunity to educate adolescents on the dangers of drug use and provide them with effective coping mechanisms and decision-making skills. Programs such as Drug Abuse Resistance Education (DARE) have been implemented in many schools to help prevent drug use among adolescents. These programs can effectively reduce the likelihood of drug use among students, improve their knowledge of the risks associated with drug use, and teach them important refusal skills [14-16].

Community awareness campaigns: Community awareness campaigns are important in raising awareness about the dangers of drug use and reducing the stigma associated with addiction. These campaigns can be targeted at parents, community leaders, and adolescents to provide information and resources on prevention and treatment. They can help promote healthy behaviors and provide individuals with the tools to identify and address potential drug problems. Community awareness campaigns can also help to increase access to treatment and support services for those struggling with addiction [3,11].

Access to Treatment and Support Services

Substance abuse treatment programs: Access to evidence-based treatments is critical in addressing adolescent substance use disorders. Substance abuse programs offer various treatment options, including behavioral therapy, medication-assisted treatment, and peer support. Behavioral therapy involves working with a therapist to identify and modify behaviors that contribute to substance use, while medication-assisted treatment uses medication to manage withdrawal symptoms and cravings. Peer support programs, such as 12-step programs, can also provide a supportive community for adolescents in recovery [14,15].

Mental health services: Adolescents with underlying mental health issues are at a higher risk of developing substance use disorders and experiencing drug overdoses. Mental health services such as counseling and therapy can help address these underlying issues and reduce the risk of drug use and overdose. Therapists can work with adolescents to address anxiety, depression, trauma, and other mental health conditions that may contribute to substance use [8,16,17].

Support groups and peer networks: Support groups can provide crucial support to adolescents in recovery, reducing the risk of relapse and overdose. These groups offer a sense of community and understanding that can be difficult to find elsewhere. Peer networks can also provide a safe and supportive space for adolescents to discuss their experiences and share coping strategies with others who have gone through similar struggles. Additionally, peer networks can serve as a source of accountability, motivating to stay sober and make healthy choices [18,19].

Policy Changes and Regulations

Prescription drug monitoring programs: Implementing prescription drug monitoring programs can help reduce the availability of prescription drugs for misuse. These programs monitor prescribing patterns and identify potential misuse or abuse of prescription drugs, which can aid in preventing addiction and overdose deaths [20].

Restrictions on opioid prescribing: The regulation of opioid prescriptions has been identified as an effective strategy to reduce the availability of these drugs for misuse and prevent overdose deaths. The implementation of prescribing regulations can limit the prescription of opioids to patients who truly require them for pain management, reducing the risk of opioid addiction and subsequent overdose deaths [21-23].

Harm reduction approaches: Harm reduction approaches, such as the distribution of naloxone and the establishment of safe injection sites, are innovative strategies to reduce the harm associated with drug use and prevent overdose deaths. Naloxone is an overdose-reversal medication that can be administered to individuals experiencing an opioid overdose. Establishing safe injection sites provides individuals with a safe and supervised environment to use drugs, reducing the risk of overdose and the spread of infectious diseases [24].

Effectiveness of prevention strategies

Overview of Studies and Data on Prevention Strategies

A wealth of research has been conducted on adolescent drug use and overdose prevention strategies. Multiple studies have evaluated various interventions, including but not limited to school-based prevention programs, community awareness campaigns, substance abuse treatment programs, and policy changes and regulations. These interventions have been assessed using a range of methodologies, such as randomized controlled trials, quasi-experimental designs, and observational studies [25].

In recent years, a growing body of literature has evaluated the effectiveness of prevention strategies for adolescent drug use and overdose. These studies have provided valuable insights into the efficacy of different interventions and have identified areas where additional research is needed [6,13,15,18,22,26]. For example, some studies have found that school-based prevention programs can reduce substance use and lower the risk of overdose among adolescents. Similarly, community awareness campaigns have been shown to increase knowledge about the dangers of drug use and encourage healthy behaviors. Substance abuse treatment programs effectively reduce drug use and improve health outcomes for adolescents with substance use disorders. In addition, policy changes and regulations, such as prescription drug monitoring programs and restrictions on opioid prescribing, have been associated with reductions in opioid-related overdose deaths among adolescents [6,15,16,22].

Despite the progress in evaluating prevention strategies for adolescent drug use and overdose, there are still significant gaps in our understanding of how best to prevent these tragedies. Additional research is needed to determine the most effective interventions for specific populations, how to implement these interventions on a larger scale, and how to sustain positive outcomes over time. Nevertheless, the current body of research provides an important foundation for developing evidence-based prevention strategies and policies that can help combat the devastating effects of adolescent drug overdoses [4,6,27].

Evaluation of the Effectiveness of Different Prevention Strategies

Evaluating the effectiveness of different prevention strategies has revealed that various approaches can effectively reduce drug use and prevent adolescent overdose deaths. For instance, school-based prevention programs and community awareness campaigns effectively reduce adolescent drug use. Furthermore, substance abuse treatment programs have also effectively reduced drug use and improved adolescent health outcomes [5,9,11,18,28,29].

Moreover, policy changes and regulations have been implemented to curb the availability of prescription drugs for misuse and reduce the risk of overdose. Prescription drug monitoring programs and restrictions on opioid prescribing are examples of policies that have effectively prevented overdose deaths among adolescents. A combination of prevention strategies may be necessary to achieve the best outcomes in reducing drug use and preventing adolescent overdose deaths [23].

Discussion of Limitations and Challenges of Prevention Efforts

Although prevention strategies have demonstrated efficacy in reducing drug use and preventing overdose deaths among adolescents, several limitations and challenges hinder their effectiveness. Principally, limited access to treatment and support services, especially in underserved communities, creates significant obstacles for prevention efforts. Addressing this challenge requires comprehensive policies and initiatives to improve access to mental health services and substance abuse treatment programs and increase resources for community-based support networks.

Furthermore, there is a crucial need for more research to identify the most effective prevention strategies and their optimal implementation. While several prevention approaches have been evaluated, further studies are necessary to assess their long-term effectiveness, scalability, and cultural appropriateness. Additionally, research efforts should focus on developing prevention programs tailored to specific populations and account for the diverse factors influencing adolescent drug use and overdose.

Finally, the ongoing opioid epidemic and the emergence of new synthetic drugs present significant challenges to prevention efforts. In response, prevention strategies should prioritize harm reduction approaches, such as expanding access to overdose-reversal medication and increasing community awareness of the risks associated with drug use. Comprehensive policy changes, such as prescription drug monitoring programs and restrictions on opioid prescribing, can also play a crucial role in curbing the opioid epidemic and preventing overdose deaths.

## Conclusions

Adolescent drug overdose deaths are a serious public health concern. Risk factors for overdose deaths include common substances involved in overdose deaths, underlying mental health conditions, peer pressure and social influences, and the lack of parental supervision and support. Effective prevention strategies include education and awareness programs, access to treatment and support services, and policy changes and regulations. Evaluations suggest that prevention strategies such as school-based community awareness campaigns, substance abuse treatment programs, and policy changes and regulations can effectively reduce adolescent drug use and overdose deaths. Future research efforts should focus on identifying the most effective prevention strategies for different populations and how to implement these strategies on a larger scale. Additionally, there is a need for more research on the long-term outcomes of prevention programs and the impact of policy changes and regulations on overdose deaths. Policy efforts should focus on increasing access to treatment and support services, implementing harm reduction approaches, and addressing the root causes of drug use among adolescents. Adolescent drug overdose deaths have a devastating impact on families and communities. Continued efforts to prevent and reduce these deaths are essential to promoting the health and well-being of adolescents. By implementing effective prevention strategies and addressing the underlying causes of drug use, we can help reduce the risk of overdose deaths and promote healthier communities for all.
